# Biosensors Coupled with Signal Amplification Technology for the Detection of Pathogenic Bacteria: A Review

**DOI:** 10.3390/bios11060190

**Published:** 2021-06-09

**Authors:** Fengchun Huang, Yingchao Zhang, Jianhan Lin, Yuanjie Liu

**Affiliations:** 1 Institute of Process Engineering, Chinese Academy of Sciences, Beijing 100190, China; huangfengchun@ipe.ac.cn; 2 College of Information and Electrical Engineering, China Agricultural University, Beijing 100083, China; s20203081467@cau.edu.cn (Y.Z.); jianhan@cau.edu.cn (J.L.)

**Keywords:** foodborne pathogens, biosensor, signal amplification, food safety

## Abstract

Foodborne disease caused by foodborne pathogens is a very important issue in food safety. Therefore, the rapid screening and sensitive detection of foodborne pathogens is of great significance for ensuring food safety. At present, many research works have reported the application of biosensors and signal amplification technologies to achieve the rapid and sensitive detection of pathogenic bacteria. Thus, this review summarized the use of biosensors coupled with signal amplification technology for the detection of pathogenic bacteria, including (1) the development, concept, and principle of biosensors; (2) types of biosensors, such as electrochemical biosensors, optical biosensors, microfluidic biosensors, and so on; and (3) different kinds of signal amplification technologies applied in biosensors, such as enzyme catalysis, nucleic acid chain reaction, biotin-streptavidin, click chemistry, cascade reaction, nanomaterials, and so on. In addition, the challenges and future trends for pathogenic bacteria based on biosensor and signal amplification technology were also discussed and summarized.

## 1. Introduction

In recent years, foodborne disease has become the most important food safety issue worldwide; it also poses a global threat to human beings. The outbreak of foodborne diseases is not only a threat to people’s health but also causes immeasurable economic losses. Most foodborne diseases are caused by different kinds of pathogenic microorganisms such as viruses, actinomycetes, fungi, parasites, and bacteria. Of these, bacteria are one of the most prevalent pathogens; as reported by Daniel Dewey, foodborne diseases caused by bacteria accounted for 47% of total foodborne diseases in 2009–2015 in the U.S. [1]. At present, pathogenic bacteria mainly include *Escherichia coli, Salmonella typhimurium, Listeria monocytogenes, Vibrio parahaemolyticus, Vibrio cholerae, Staphylococcus aureus*, and *Bacillus cereus* [2]. Humans can be infected with pathogenic bacteria by contact with contaminated water, foods, and air, leading to serious health problems [3,4]. It has been noted that almost one-third of global mortality is caused by bacterial infections [5]. Furthermore, diarrhea kills around 520,000 children every year [6]. Therefore, preventing foodborne pathogens from contaminated food and reducing and avoiding the occurrence of foodborne diseases are key projects that need to be addressed in the field of food safety. At present, the prevention of foodborne diseases is mainly achieved by cleaning, using safe drinking water and raw materials and separating raw and cooked foods. However, some processed foods will be inevitably contaminated by pathogenic bacteria in their packaging, transportation, and sales procedures. It is impossible to prevent contamination using the above-mentioned methods. Thus, the development of rapid technology to achieve the early screening of pathogenic bacteria will effectively reduce the occurrence of foodborne diseases, and this has significant value for practical applications in food safety.

To date, different methods have been studied and employed to detect pathogenic bacteria, such as bacterial cultivation, polymerase chain reaction (PCR), loop-mediated isothermal amplification (LAMP), enzyme-linked immunosorbent assay (ELISA), and immunochromatographic assay (ICA). Culture counting, as the current gold standard for pathogenic bacteria detection, has the advantages of simple equipment, a low cost, easy operation, and high accuracy [7]. However, this method cannot meet the requirements of rapid and on-site detection because it needs a long time (more than 24 h or even a week) and complex operation in laboratory. With its advantages of higher sensitivity and a shorter detection time, the PCR and LAMP are increasingly recommended for pathogenic bacteria detection [8,9,10], but its further application has been limited due to the shortcomings of the expensive equipment required, complex nucleic acid extraction process, and false positive results caused by aerosol pollution. In addition, the immunological methods based on the specific recognition between the antigen and antibody (mainly including ELISA [11] and ICA [12]) are increasingly applied for the detection of pathogenic bacteria. To date, low stability, cross-reactions, non-specific adsorption, and false-positive results are the challenges that the immunoassay methods need to overcome, even though they exhibit low-cost and high-throughput performance. In summary, the traditional methods still have some shortcomings that need to be solved urgently, even though they have their own advantages. In addition, the complex food background and the ultra-low concentration of bacteria in the screening samples causes great challenges for the rapid and sensitive detection of pathogenic bacteria. Therefore, to achieve the required performance, increasing numbers of scientists have explored new technologies to meet the growing demand for food safety. Thus, biosensors, as emerging technologies, have received widespread attention, providing the benefits of highly selective, reliable, and rapid pathogenic detection.

## 2. Biosensors

### 2.1. Development, Concept, and Principle of Biosensors

In 1962, an enzyme-based biosensor was reported for the first time by Professor Clark on the Annals of the New York Academy of Sciences [13]. In this biosensor, the glucose oxidase (GOx) was modified on the surface of the oxygen electrode through a dialysis membrane; then, the concentration of glucose would be related to the decrease of oxygen. Thus, the correlation between the enzyme activity of the GOx and glucose concentration was achieved. It is worth noting that this study created a new chapter for the development of biosensors. Subsequently, the first glucose meter based on the GOx biosensor was developed by the American company of Yellow Springs instrument (YSI) in 1975, commercializing the biosensor invented by Professor Clark [13]. In recent years, with the development and cross-integration of biology, analytical chemistry, nanomaterials science, electronics, informatics, and micro processing, biosensors have been developed rapidly, providing a highly sensitive and selective analysis method for life science research and clinical experiments, and it is also widely used in food safety, environmental protection, and medical research.

**Figure 1:**
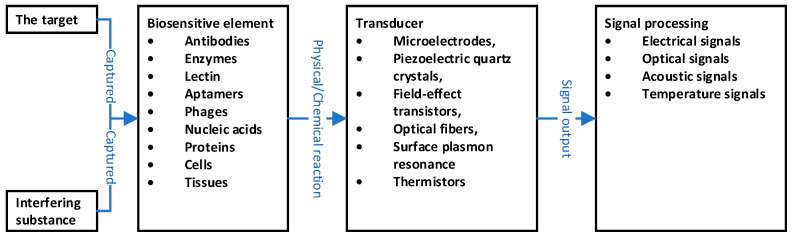
Principles of biosensors.

The concept of biosensors was defined by the International Union of Pure and Applied Chemistry (IUPAC) as follows: “a biosensor is an independent integrated device, which can directly combine a kind of transducer with a kind of biometric element, so that it can specifically analyze the target quantitatively or semi-quantitatively” [14]. From this definition, the principle of biosensors is to transfer the physical or chemical reactions between a bio-sensitive element (mainly including antibodies, enzymes, lectin, aptamers, phages, nucleic acids, proteins, cells, and tissues) and targets to transducers (including microelectrodes, piezoelectric quartz crystals, field-effect transistors, optical fibers, surface plasmon resonance and thermistors, etc.). The transducer converts the received physicochemical reaction signals into measurable signals (such as electrical signals, optical signals, acoustic signals, temperature signals, etc.) and outputs them for presentation. The measured signals can indirectly reflect the concentration of the target [15] (as shown in Figure 1). Here, the bio-sensitive element can recognize specific targets and produce a binding reaction that has high specificity. This determines the specificity of the biosensor, thus playing a very important role. At the same time, the diversity of transducers makes it possible for different analysis fields to choose biosensors according to their own needs.

### 2.2. Types of Biosensors

With the development and application of sensors in multiple disciplines, many biosensors in different forms have been derived. At present, the biosensors can be classified according to its bio-sensitive element and transducer. Regarding the bio-sensitive element, it can be classified into enzyme, DNA, tissue, and immunosensor biosensors. According to the transducers used, we can classify biosensors into electrochemical, optical, piezoelectric, thermal, and acoustic sensors [16]. At the same time, with the development of microfluidic technology, a new type of microfluidic biosensor has been developed, which has advantages in automation and miniaturization. In recent years, electrochemical biosensors, optical biosensors, and microfluidic biosensors have attracted great attention and become a research hotspot.

#### 2.2.1. Electrochemical Biosensors

Electrochemical biosensors are an important branch of biosensors with the longest history, the widest application range, and the best vitality. Electrochemical biosensor is a typical sensing device that transduces the biochemical events to electrical signals [17]. Generally speaking, the electrochemical biosensor fixes biological recognition elements (such as antibodies and aptamers) on the surface of certain electrodes by biological modification. The fixed biological sensitive elements will recognize the target molecules in the solution and produce a specific binding reaction. The binding reaction is converted into an electrical signal through the electrode, achieving quantitative or semi-quantitative detection. Electrochemical biosensors can be classified into amperometric, impedance, potential, and capacitance types. Among them, amperometric and impedimetric biosensors are most widely used in the detection and analysis of molecules because of their high sensitivity.

#### 2.2.2. Impedimetric Biosensors

Impedance in a circuit is used to measure the block level encountered by an alternating current. It is the general name for resistance, inductance, and capacitance. In an electrochemical system, impedance is an important parameter to describe the interfacial properties of electrode and electrolyte [18]. An impedimetric biosensor is a kind of electrochemical detection method to quantitatively detect a target molecule. It applies a small amplitude of alternating current (AC) voltage on the electrode surface to produce a disturbance, and the difficulty of electron transfer in the solution is measured [19]. In this kind of method, the microelectrode is commonly used in impedance biosensors. According to whether the microelectrode is modified with a biometric element, it can be categorized as a faradaic and non-faradaic impedimetric biosensor. For example, in electrode-modified impedimetric biosensors, Fe(CN)_6_^3-/4-^ is often used as an auxiliary probe for detection. When the electrode is modified with a biometric element with poor conductivity, it will hinder the diffusion of Fe(CN)_6_^3-/4-^ to the electrode surface, resulting in increasing impedance. When the target molecule reacts with the recognition element, it will lead to a further increase in impedance. Therefore, the quantities of the molecules to be measured can be calculated according to the change of impedance. In non-electrode modified impedimetric biosensors, an enzyme is often introduced for the enzymatic reaction, which leads to a change in the ion concentration in the detection solution, thus causing a change of impedance. A linear relationship can be established between the impedance change and the concentration of the target molecule to achieve the detection.

**Figure 2:**
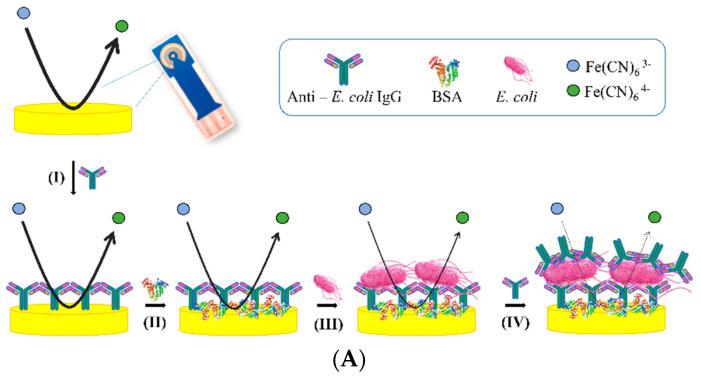

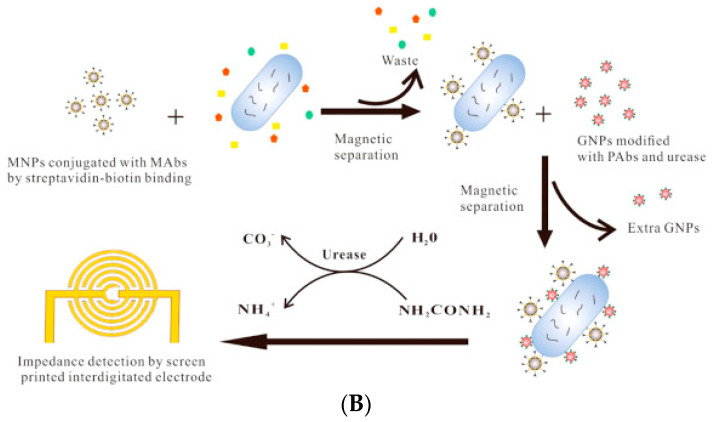
Application of an impedimetric biosensor in foodborne pathogen detection: (**A**) electrode-modified impedimetric biosensor for the detection of *E. coli* [25]; (**B**) electrode-modified-free impedimetric biosensor for the detection of *L. monocytogenes* [26].

The impedimetric biosensor is also widely used in the rapid and sensitive detection of foodborne pathogens [20,21,22,23]. For example, Farka et al. (2016) immobilized an antibody on two gold electrodes of a screen-printed electrode by glutaraldehyde-activated cysteamine [24]. The immobilized antibody can be used to capture *S. typhimurium* on the electrode. The impedance change between the electrodes is used to detect the target bacteria. The procedure can be finished in 20 min, and the detection limit can be as low as 1 × 10^3^ CFU/mL. Cimafone et al. (2020) reported a method for *E. coli* quantitative detection using an impedimetric biosensor based on a modified screen-printed electrode [25]. The *E. coli* antibody was fixed on the electrode by modifying where the bacteria was captured. By measuring the electrochemical impedance spectrum of Fe(CN)_6_^3−/4−^ after each modification and binding, amounts of *E. coli* in drinking water as low as 3 × 10^1^ CFU/mL can be detected within 1 h (as shown in Figure 2A). Wang et al. (2017) formed a double antibody sandwich structure between immunomagnetic beads, *L. monocytogenes*, and colloidal gold particles modified with urease and target bacteria antibodies [26]. Then, they redissolved the double antibody sandwich complex in urea solution with poor conductivity. Since urease on the complex can catalyze urea to produce ions (ammonium ions and carbonate ions), it can be detected by the decrease of impedance caused by the increase of ion concentration. The impedimetric biosensor can detect *L. monocytogenes* with a concentration higher than 1.6 × 10^3^ CFU/mL rapidly without modifying the electrode (as shown in Figure 2B).

#### 2.2.3. Amperometric Biosensors

Amperometric biosensors measure changes in electrical current. Amperometry involves increases or decreases in signal as the result of oxidation or reduction reactions involving the analyte, driving a current response that corresponds to the analyte concentration. The signal is detected using an electrode, which is held at a chosen potential to facilitate the transfer of electrons [27]. To date, amperometric biosensors mainly depend on the cyclic voltammetry (CV) method, which is often used to acquire the parameters of the reaction of an electrode and the mechanism and kinetics of the reaction process. By controlling the electrode potential, the redox reaction can take place alternately on the electrode. The heights of the redox peaks corresponding to the triangular waveform obtained by scanning different electroactive substances on the electrode are different. There is a positive correlation between the height of the redox peak and the substance to be measured. By analyzing the redox curve, the material to be measured on the electrode can be quantitatively analyzed.

**Figure 3:**
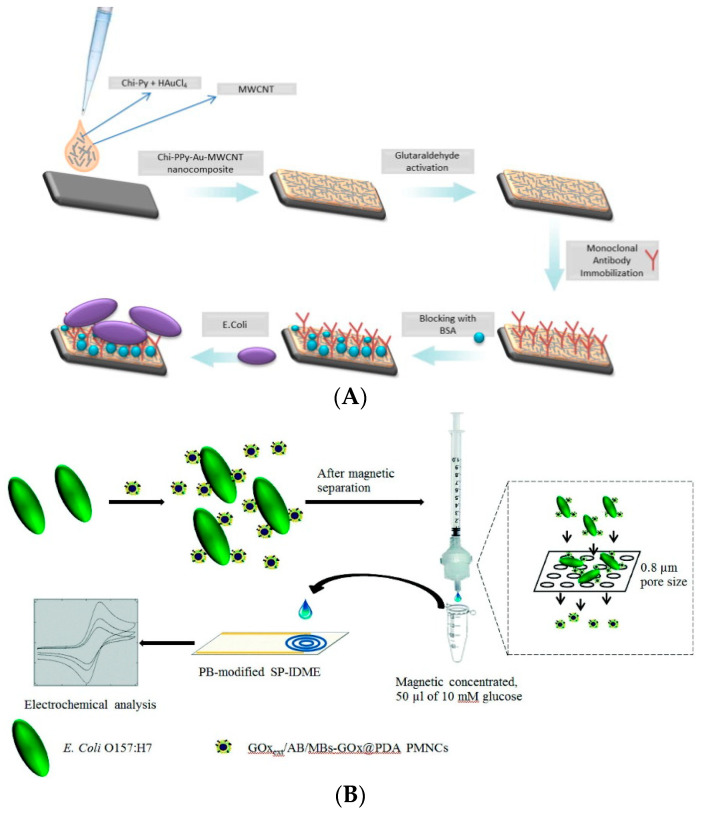
Application of amperometric biosensor in foodborne pathogens detection: (**A**) Electrode modified amperometric biosensor for detection of *E. coli* O157:H7 [31]; (**B**) Electrode modified-free amperometric biosensor for detection of *E. coli* O157:H7 [32]. MWCNT: multi-walled carbon nanotube.

In recent years, amperometric biosensors based on cyclic voltammetry have been combined with immune technology and achieved a reduction of the detection time and limit. Therefore, they are widely applied in the rapid and sensitive detection of multi-target molecules and foodborne pathogenic bacteria [28,29]. For example, Matta et al. (2018) fixed a “dip-coating rod” with nano-magnetic beads modified with carbohydrate ligands to extract bacteria from complex samples and then determined the concentration of extracted bacteria by cyclic voltammetry [30]. This method can detect 10^2^ CFU/mL *E. coli* O157:H7, *S. enteritidis,* and *L. monocytogenes* within 30 min. Guner et al. (2017) developed an amperometric immunosensor with high sensitivity for the detection of *E. coli* [31]. By labeling polypyrene/colloidal gold/carbon nanotubes/chitosan on the graphite electrode, a monoclonal antibody that can recognize *E. coli* O157:H7 was immobilized on the electrode surface. The bacteria become trapped on the surface and lead to redox reaction, whose peak can be determined by cyclic voltammetry. In this way, the concentration of the bacteria can be inferred. The method can detect *E. coli* O157:H7 at amounts as low as 30 CFU/mL (Figure 3A). Xu et al. (2017) modified the upper layer of magnetic nanoparticles with GOx, dopamine, colloidal gold nanoparticles, and antibodies that specifically recognize the target molecules and combined them with *E. coli* O157:H7 through antibody recognition to form a complex [32]. The unbound magnetic nanoparticles were removed using a syringe with a filter membrane (with a 0.8-micron aperture). The collected complex was resuspended in glucose solution and was catalyzed by GOx to produce a redox reaction. There is a linear relationship between the current change caused by the reaction and the concentration of *E. coli* O157:H7. The detection limit of the target bacteria is 10^2^ CFU/mL by cyclic voltammetry (Figure 3B).

#### 2.2.4. Optical Biosensor

An optical biosensor is used to analyze and detect a target using the absorption, fluorescence, refraction, and reflection characteristics of light. Optical biosensors have the advantages of fast detection speed, low detection cost, strong anti-interference ability, and on-site application, and so they are widely used in environmental monitoring, clinical medicine, food safety, and other fields [33]. According to the different properties of light, optical biosensors can be divided into colorimetric biosensors, fluorescent biosensors, surface-enhanced Raman spectroscopy biosensors, and surface plasmon resonance biosensors. Of these, colorimetric and fluorescent biosensors are widely used as optical biosensors because of their advantages of easy interpretation and high sensitivity [34,35,36].

#### 2.2.5. Colorimetric Biosensor

Colorimetric analysis is an analytical technique for the quantitative detection of analytes according to the color changes caused by the reaction between analytes of different concentrations and experimental reagents [37]. The development of colorimetric analysis technology has been in two stages: visual colorimetry and photoelectric colorimetry. For visual colorimetry, the target concentration is mainly judged by observing the color that appears in the detecting reaction with the naked eye. The experimental results depending on visual observations are not always reliable, so the accuracy of this analysis method is low. Thus, a method of photoelectric colorimetry based on visual colorimetry has gradually developed. In photoelectric colorimetry, the absorbance of standard solution in different concentrations is measured to draw a standard curve. Then, quantitative analysis can be carried out according to the absorbance of the substance to be measured. Compared with visual colorimetry, photoelectric colorimetry eliminates the error caused by humans and greatly improves the accuracy of the experimental results. At present, the commonly used detection instruments of photoelectric colorimetry mainly include a spectrophotometer, ultraviolet-visible spectrophotometer, and microplate reader.

**Figure 4:**
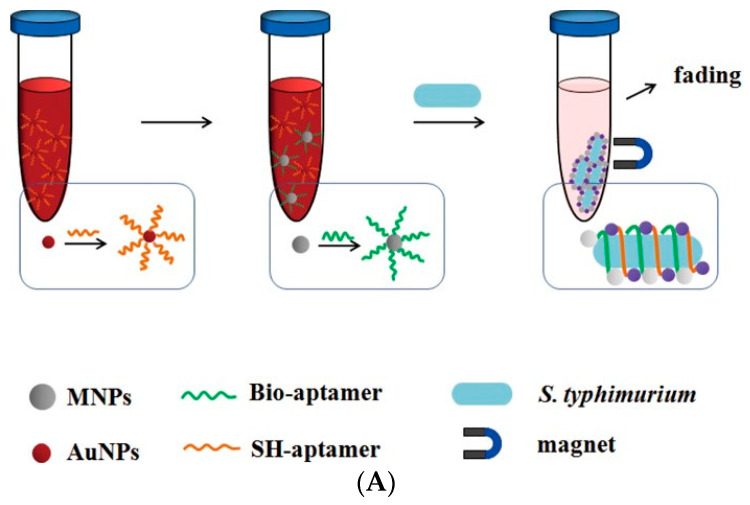

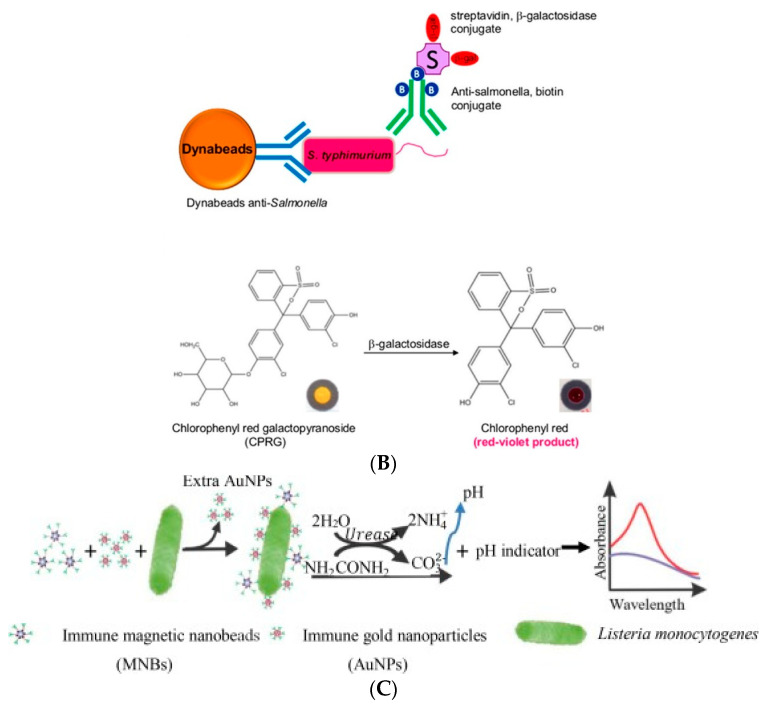
Application of colorimetric biosensor in foodborne pathogen detection: (**A**) [38] and (**B**) [39] *S. typhimurium* detection; (**C**) *L. monocytogenes* detection [40] using coloration by pH indicator.

The colorimetric biosensor based on photoelectric colorimetric analysis is also often used in the detection of foodborne pathogens. For example, Duan et al. (2016) dissolved aptamer-modified nano-magnetic beads and colloidal gold particles in dark-red colloidal gold solution [38]. Then, *S. typhimurium* was added to form a sandwich complex with immunomagnetic beads and colloidal gold particles through the aptamer. Under the effect of magnetic separation, the complex agglomerates in the solution, meaning that the original dark red colloidal gold solution fades into light pink. Different concentrations of target bacteria cause different degrees of color changes, which can be used for the quantitative analysis of *S. typhimurium*. The detection limit can reach 10 CFU/mL, achieving the ultra-sensitive detection of foodborne pathogens (Figure 4A). Srisa-Arta et al. (2018) also formed a double antibody sandwich complex of *S. typhimurium*, magnetic nanoparticles, and β-galactosidase-labeled nanomaterials through an antigen–antibody reaction [39]. After being dissolved in the yellow chlorophenol red-β-D-galactopyranoside solution, its color changes from yellow to red. According to the color change, the concentration of *S. typhimurium* can be determined. The method can detect target bacteria at amounts as low as 10^2^ CFU/mL (Figure 4B). Chen et al. (2018) combined magnetic nanoparticles and colloidal gold particles with *L. monocytogenes* to form a double antibody sandwich complex, in which the colloidal gold particles were also modified with urease [40]. The urease can catalyze the substrate of urea and cause a change of pH. By adding a pH indicator, quantitative analysis of the target bacteria can be achieved by measuring the absorbance of the color change in the solution. This method can detect amounts of *L. monocytogenes* as low as 10^2^ CFU/mL (Figure 4C).

#### 2.2.6. Fluorescence Biosensor

A fluorescence biosensor is a biosensor based on the fluorescent properties of substances. At present, fluorescence spectroscopy and fluorescence energy resonance transfer methods are most commonly used. In brief, the principle of fluorescence spectroscopy is that different substances will emit different wavelengths of the fluorescence spectrum after absorbing the energy of light under UV irradiation, and the intensity is related to the concentration. Fluorescence spectrometry mainly uses this correspondence to determine the concentration of specific substances [41]. In the fluorescence resonance energy transfer method, the emission spectrum of one fluorescent substance overlaps with the absorption spectrum of another substance. The latter emits lights after absorbing the energy of the former. By measuring the emission spectrum of the latter, the concentration of the former can be obtained [42]. Quantum dots (QDs), fluorescent dyes, and nanomaterials with fluorescent properties are often used in fluorescence spectroscopy, while a combination of nanomaterials that can experience energy transfer is commonly used in fluorescence resonance energy transfer. Fluorescent biosensors have the advantages of simple operation, high sensitivity, and non-contact detection. They are widely studied and applied in environmental monitoring, medical diagnosis, and food safety fields [43,44,45].

**Figure 5:**
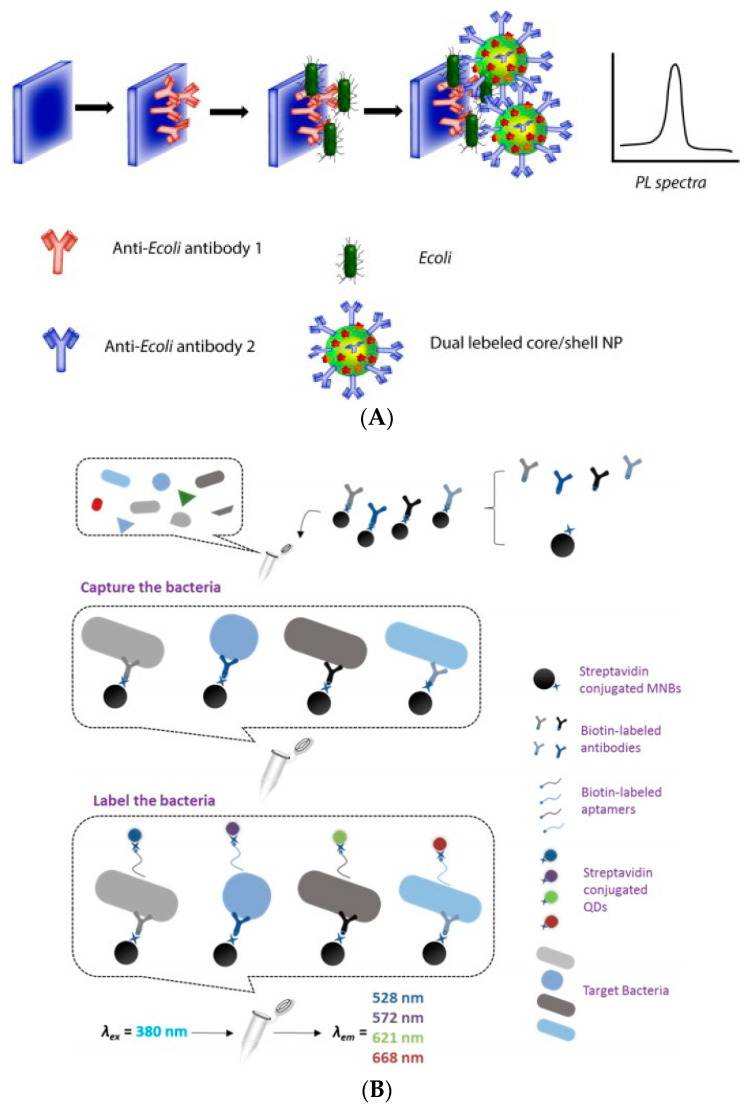
Application of fluorescence biosensor in foodborne pathogen detection: (**A**) *E. coli* detection [46]; (**B**) simultaneous detection of multiple foodborne pathogens [48].

Fluorescent biosensors are often used for the rapid and sensitive detection of foodborne pathogens, making a great contribution to the early screening. Krishnan et al. (2014) developed a new fluorescent biosensor based on fluorescent dye and Ag@Si Core shell nanoparticles modified with antibodies against *E. coli* [46]. The sensor was modified with a quartz glass plate with the antibody of the target bacteria. After *E. coli* was captured, the Ag@Si core–shell nanoparticles were combined through the antibody. There was a positive correlation between the fluorescence intensity of dyes on nanoparticles and *E. coli*. By measuring the fluorescence intensity, amounts of *E. coli* as low as 5 CFU/mL could be detected. In the detection of foodborne pathogens, this method has the advantage of high sensitivity (Figure 5A). In fluorescent biosensors, QDs are one of the most studied and promising nanomaterials, which are also used in the detection of foodborne pathogens. For example, Kuang et al. (2013) carried out a quantitative analysis of *S. typhimurium* by modifying nano-magnetic beads and QDs with an antibody of *S. typhimurium* [47]. The results showed that the fluorescent biosensor based on QDs can detect 5 × 10^2^ CFU/mL in 30 min. This method has the advantages of simple operation, high sensitivity, and short detection time. In addition, QDs with different fluorescence colors have different emission wavelengths, meaning that we can use this property to detect multiple foodborne pathogens simultaneously. For example, Xu et al. (2015) used QDs with different emission wavelengths (528 nm, 572 nm, 621 nm, and 668 nm) to bind with aptamers of different target bacteria [48]. Then, corresponding complexes containing the target bacteria were formed by using immunomagnetic beads labeled with antibodies. These bacteria can be detected simultaneously by measuring the fluorescence intensity of QDs on each complex. The method enables the simultaneous detection of *E. coli O157:H7*, *S. aureus*, *L. monocytogenes*, and *S. typhimurium*, and the detection limits are 8 × 10^1^ CFU/mL, 10^2^ CFU/mL, 4.7 × 10^1^ CFU/mL, and 1.6 × 10^2^ CFU/mL, respectively (Figure 5B).

#### 2.2.7. Microfluidic Biosensor

**Figure 6:**
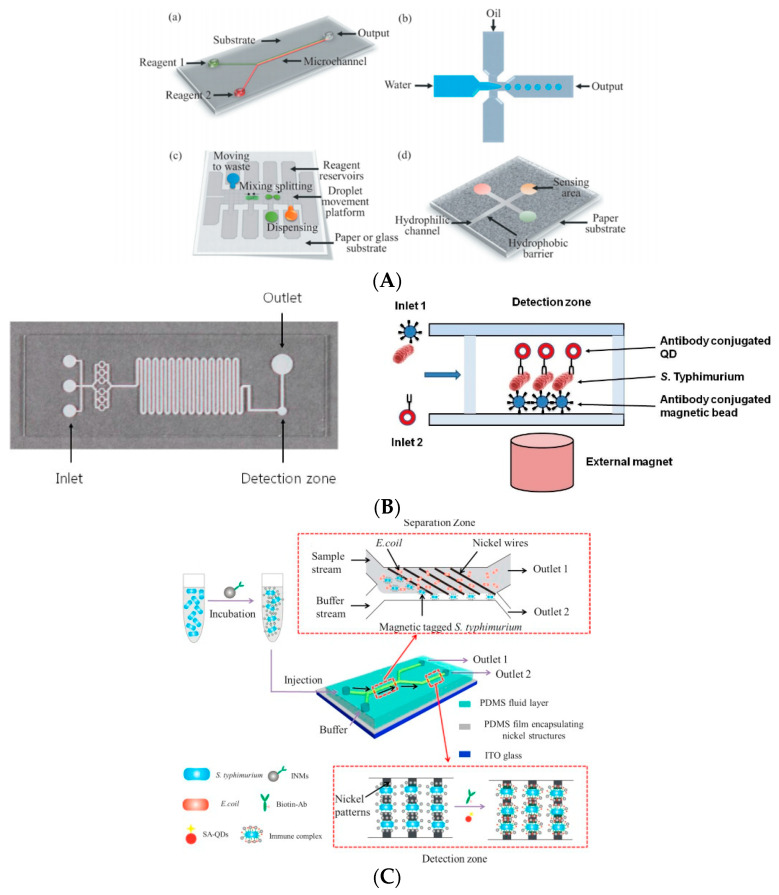
Microfluidic biosensor: (**A**) different kinds of microfluidic chips [53]. Application in foodborne pathogen detection: (**B**) *S. typhimurium* detection [52]; (**C**) *E. coli* detection [55].

The microfluidic chip, also known as a lab-on-a-chip, is a technology that uses channels with sizes of tens to hundreds of microns to manipulate and process micro-volume samples. In recent years, the microfluidic biosensor has been developed by combining this technology with biochemical analysis technology. The sensor can integrate the analysis process (sample pretreatment, sample separation, biochemical reaction, and real-time quantitative analysis) on a single microfluidic chip [49,50]. At present, silicon, glass, quartz, polydimethylsiloxane (PDMS), and paper-based materials are mainly used in the fabrication of microfluidic chips. In addition, the fabrication technologies of chips mainly include lithography, etching, hot pressing, and molding [51,52]. Microfluidic biosensors for detection are mainly based on several types of chips: a continuous flow microfluidic chip, micro droplet flow microfluidic chip, digital flow microfluidic chip, and paper-based microfluidic chip [53] (Figure 6A). Due to the complexity of the sample matrix, microfluidic chips are often combined with different types of detectors to meet the requirements of the separation and biochemical analysis of different types of targets. According to the detectors mounted on chips, this category of biosensors can be divided into microfluidic optical, electrochemical, and chromatographic [54].

In view of its advantages of miniaturization, automation, portability, low cost, short detection time, and high-throughput parallel detection, microfluidic biosensors are widely used in the field of rapid and sensitive detection, including the early screening and rapid detection of foodborne pathogens [56]. For example, Alves et al. (2019) developed a microfluidic biosensor based on an FEP-Teflon capillary and fluorescent materials and combined this with smartphones to detect *E. coli*. Its detection limit can reach as low as 10^3^ CFU/mL [57]. The innovative combination of a microfluidic chip and smartphone provides an important means for on-site detection. Dastider et al. (2013) fabricated a microfluidic chip based on impedance detection by embedding interdigital array microelectrodes into PDMS and glass-bonded channels [58]. In this method, an antibody that can specifically recognize *E. coli* was modified on the electrode. When the microfluid containing *E. coli* flows through the chip, the target bacteria are recognized and captured by the antibody on the electrode, resulting in the change of impedance. There is a linear correlation between the change of impedance and bacteria. The microfluidic biosensor can successfully detect amounts of *E. coli* as low as 3 × 10^2^ CFU/mL and achieves rapid, sensitive, and automatic analysis. Kim et al. (2015) designed a microfluidic chip containing a sample inlet, reaction-binding region, and detection region. In the reaction-binding region, serpentine channels are used to increase the contact between samples to improve the reaction efficiency [59]. When the antibody-modified magnetic beads flow through the serpentine channel, they are fixed and dispersed in the channel under the pull of the magnet below the channel. Then, the QDs labeled with the antibody are injected into the reaction zone to form a double antibody sandwich complex with bacteria and magnetic nanoparticles. After removing the magnetic field, the compound flows into the detection area. The fluorescence detection device connected with the chip can detect the target bacteria by measuring the fluorescence intensity of the QDs. The microfluidic chip reached the detection limit of 10^3^ CFU/mL for *S. typhimurium* (Figure 6B). The microfluidic chip developed by Guo et al. (2015) contains a target bacteria isolation region and detection region (as shown in Figure 6C) [55]. The metal material of a magnetic nickel wire is placed in the separation zone. The nano-magnetic beads modified with an antibody can be firmly grasped by the nickel wire and flow into the detection area under the magnetic force generated below the channel when passing through the separation zone. The bacteria without magnetic nanoparticles will flow out from outlet 1. A nickel module is also set in the detection area to fix the target bacteria. At the same time, the QDs with fluorescence characteristics are combined by antibodies to obtain double antibody sandwich complexes that can be used for fluorescence intensity detection. The microfluidic chip achieves the integration of the separation, enrichment, and detection, which can detect amounts of *E. coli* as low as 5.4 × 10^3^ CFU/mL.

In conclusion, biosensors have the advantages of high sensitivity, short response time, and fast detection speed. They are widely used in the detection of foodborne pathogens. At the same time, because of their shortcomings, they still need to be continuously improved. For example, in an electrochemical biosensor, a small number of targets can cause a change of the signal, thus obtaining a high sensitivity. However, for the same reason, electrochemical biosensors are vulnerable to external interference. In an impedimetric biosensor, excess ions significantly interfere with the experimental results, and the stability and accuracy of the sensor need to be improved. At present, the impedance-type and amperometric-type biosensors need to use a large electrochemical workstation to implement the final electrical signal output and processing. This is a big problem for the rapid on-site detection of foodborne pathogens. It is necessary to develop microminiaturized electrochemical means to broaden their application prospects. The sensitivities of optical biosensors based on colorimetric analysis are varied due to the different extinction coefficients of diverse substances. It is a great challenge to improve the sensitivity of colorimetric analysis in general cases. The signal of a fluorescent biosensor comes from the fluorescence property of a substance. However, fluorescence is easily quenched under certain conditions (such as a specific temperature or pH), which raises strict requirements for the reaction conditions of fluorescent biosensors. The development of a microfluidic chip has led to great progress for biosensors in terms of miniaturization, automation, and integration, providing great help for the on-site detection of foodborne pathogens. However, due to the micro scale of microfluidic channels, there are challenges in the analysis of complex samples. For example, the channel’s non-specific adsorption of the sample and the blockage of the channel are problems that are difficult to solve. Therefore, to address current issues, we still need to develop biosensors in combination with more advanced technologies to improve their performance in practical detection.

## 3. Signal Amplification Technology in Biosensors

It is the basic principle of biosensors to quantitatively analyze the target by using the positive correlation between the target concentration and the detection signal. However, in practical detection applications, we often face the problem that the signal conversion and output cannot be triggered due to the low concentration of the target. If the concentration of the target remains unchanged, increasing the intensity of the corresponding output signal can deal with this problem. Therefore, the development of signal amplification technology to improve the detection sensitivity and detection range of biosensors has become a research hotspot. At present, the signal amplification technology used in biosensors is becoming increasingly diversified. In the same biosensor, multiple signal amplification techniques can be applied simultaneously [60]. To date, the most applied and researched signal amplification technologies are mainly based on enzyme catalysis, nucleic acid chain reaction, biotin–streptavidin (SA), click chemistry, cascade reaction, and nanomaterials [61].

### 3.1. Signal Amplification Technology Based on Enzyme Catalysis

An enzyme is a kind of protein or RNA produced by living cells that has high selectivity and catalytic performance for substrates [62]. Using the excellent properties of the enzyme to catalyze the corresponding substrate, undetectable biochemical reactions can be converted into signals for output. This is an effective way to improve the sensitivity of the detection method [63]. The construction of signal amplification technology based on enzyme catalysis can effectively improve the application prospect of biosensors. At present, the commonly used catalytic enzymes include horseradish peroxidase (HRP), GOx, invertase (int), urease, and alkaline phosphatase (ALP) [64,65].

**Figure 7:**
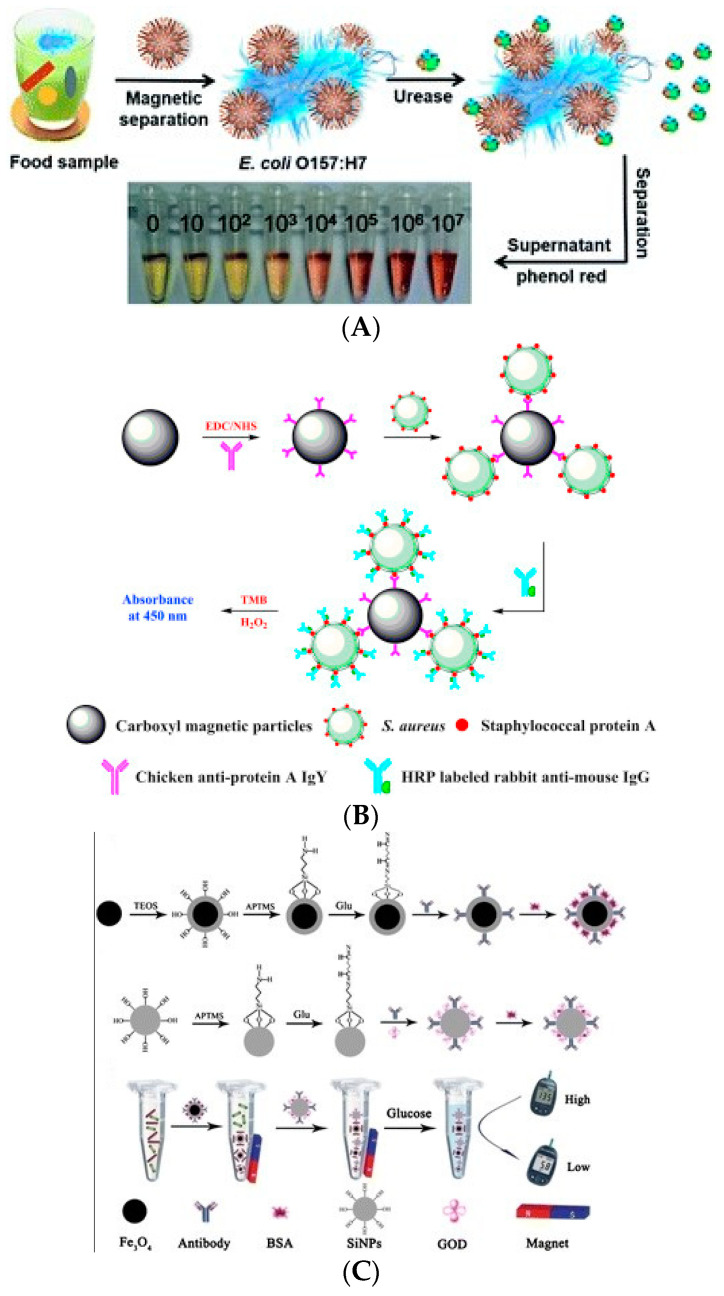
Application of enzyme-catalyzed signal amplification technology in the detection of foodborne pathogens: (**A**) *E. coli* O157:H7 detection [66]; (**B**) *S. aureus* detection [67]; (**C**) *S. typhimurium* detection [68]. HRP: horseradish peroxidase.

Signal amplification technology based on enzyme catalysis has been widely used in biosensors for the detection of foodborne pathogens. For example, Qiao et al. (2017) used magnetic nanoparticles labeled with antimicrobial peptides to capture *E. coli* O157:H7 through electrostatic and hydrophobic interactions [66]. Due to the capture of the target bacteria by magnetic nanoparticles, the binding sites of urease are blocked, which means that the supernatant contains a large amount of urease after magnetic separation. Urease can catalyze the substrate urea to produce carbonate ions and ammonium ions, which can change the pH of the solution. At this time, adding a pH indicator can change the color of the solution from yellow to purple. The quantitative analysis of *E. coli* O157:H7 can be carried out by measuring the color change with absorbance. Signal amplification technology based on enzyme catalysis can detect 12 CFU/mL *E. coli* O157:H7 in 30 min. This method proves that the urease catalytic technology can enable the signal amplification for the biosensor (Figure 7A), achieving high sensitivity in a short time. Zhang et al. (2019) labeled an antibody on nano-magnetic beads that could specifically recognize the surfactant protein-A of *S. aureus* [67]. Other protein-A sites on the surface of bacteria can recognize and bind with HRP-labeled antibody, meaning that a large amount of HRP can be bound on the surface of bacteria. The enzyme can catalyze the substrate of 3,3′,5,5′-tetramethylbenzidine (TMB) and hydrogen peroxide (H_2_O_2_) to produce color changes to achieve target detection. This method could reach the detection limit of 5 × 10^2^ CFU/mL in 90 min, and the rapid and sensitive detection of *S. aureus* was achieved (Figure 7B). Luo et al. (2017) modified an antibody that could recognize *S. typhimurium* on magnetic nanoparticles and silicon nanoparticles by amino labeling [68]. The surface of silicon nanoparticles was modified with GOx to form a double antibody sandwich structure with *S. typhimurium*. GOx was used to catalyze the decomposition of glucose, which resulted in the decrease of glucose concentration. *S. typhimurium* can be quantified by measuring the change of glucose concentration with a blood glucose meter. The sensor can detect amounts of *S. typhimurium* as low as 72 CFU/mL, which proves once again that the method based on enzyme catalysis is capable of signal amplification (Figure 7C).

### 3.2. Signal Amplification Technology Based on Nucleic Acid Chain Reaction

It is the basic principle of PCR to amplify a target DNA fragment using complementary base pairing. Inspired by this technology, scientists have developed a signal amplification method based on DNA cycle amplification: in combination with immunology, the DNA fragment is labeled with a certain signal molecule to cause it to reach the maximum load and achieve the amplification of the output signal [69]. This technology can be used in biosensors to improve their sensitivity. At present, the commonly used reactions for signal amplification based on nucleic acid chain reaction mainly include polymerase chain reaction (PCR), rolling circle amplification reaction (RCA), hybrid chain reaction (HCR), ligase chain reaction (LCR), and loop-mediated isothermal amplification (LAMP) [70].

**Figure 8:**
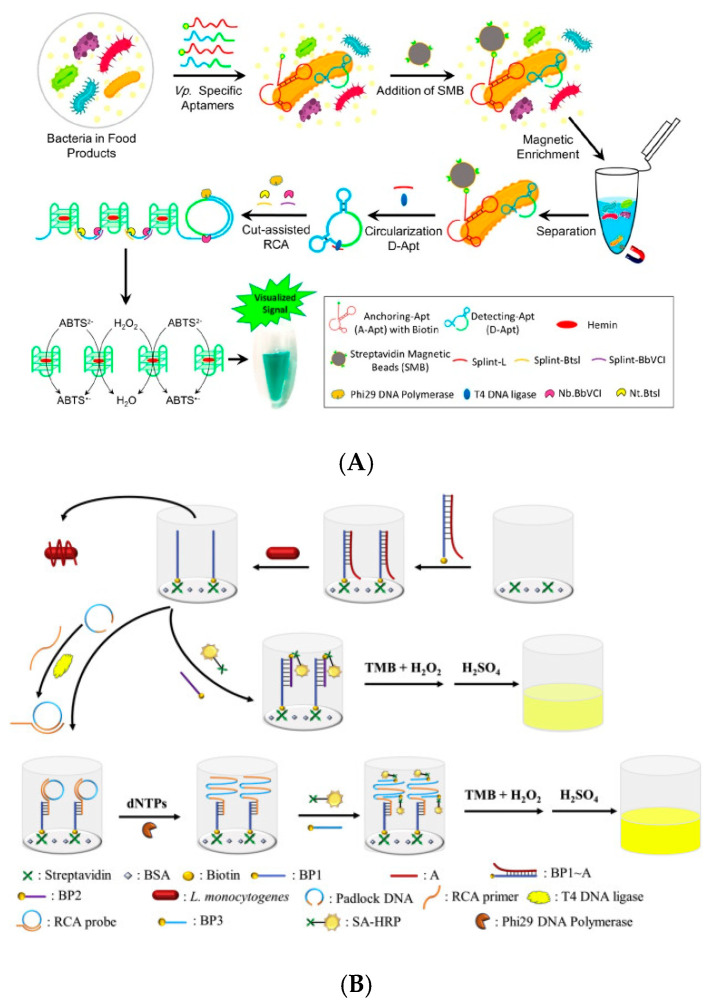
Application of nucleic acid chain reaction based on signal amplification technology in the detection of foodborne pathogens: (**A**) *V. parahaemolyticus* detection [71]; (**B**) *L. monocytogenes* detection [72]. RCA: rolling circle amplification reaction.

Signal amplification technology based on nucleic acid chain reaction has also been applied to the detection of foodborne pathogens [73,74,75,76]. For example, Tang et al. (2019) developed a simple enzyme-free signal amplification strategy based on HCR amplification technology and graphene oxide (go) for the sensitive detection of *S. aureus* [77]. Two hairpin probes (HP1 and HP) labeled with fluorescent groups were used. In the absence of the target (16S rRNA, a specific nucleic acid fragment of *S. aureus*), the two hairpin probes are adsorbed by GO through π–π stacking, resulting in the quenching of the fluorescence signal. When there is a target nucleic acid, the two hairpin probes will trigger an HCR reaction and generate a double-stranded DNA complex. In this way, the fluorescent signal can be amplified by the interaction of fluorescent groups and fluorescent dyes on double-stranded DNA to achieve the sensitive quantification of the target fragment. Under optimal reaction conditions, the detection limit of the 16S rRNA fragment of *S. aureus* can reach 50 pM. Correspondingly, the lowest detectable concentration of *S. aureus* is 4 × 10^2^ CFU/mL. Song et al. (2019) used two aptamers to capture and recognize *V. parahaemolyticus* [71]. Then, the G4 sequence, which can catalyze 2, 2′-azino-bis(3-ethylbenzothiazoline-6-sulfonic acid) (ABTS^2-^) oxidation, was generated by RCA-assisted cleavage technology. Its signal amplification effect enables the naked eye to identify the detection results. The amplification technology can detect amounts of *V. parahaemolyticus* as low as 10 CFU/mL without DNA extraction and expensive equipment, achieving ultra-sensitive detection (Figure 8A). Lv et al. (2019) carried out the amplification of a fluorescence signal using fluorescent probe-modified single-strand DNA and HCR technology [78]. In this method, a variety of antibodies were used to capture three different types of foodborne pathogens. HCR was used to implement the complementary circular amplification of single-stranded DNA to achieve the effect of loading more fluorescent probes. This technique can be used to detect three kinds of bacteria in the same sample: *E. coli* O157:H7, *S. typhimurium*, and *L. monocytogenes*. The detection limits were as low as 34 CFU/mL, 6.4 CFU/mL and 70 CFU/mL, respectively. Zhan et al. (2020) developed a signal amplification method for the sensitive detection of *L. monocytogenes* by combining ELISA with RCA technology [72]. A large amount of HRP was loaded on the detection complex by RCA rolling amplification. HRP can catalyze the substrate of TMB to enhance color reaction. The detection limit was 4.6 × 10^2^ CFU/mL (Figure 8B).

### 3.3. Signal Amplification Technology Based on Biotin–SA

Biotin, also known as vitamin H or coenzyme R, is a water-soluble vitamin. Avidin is a glycoprotein composed of four subunits that can be extracted from egg white. Streptavidin (SA) is a protein secreted by *Streptomyces* with similar biological characteristics to avidin. There are four subunits on avidin, which determines that one avidin molecule can bind to four biotin molecules. SA’s molecular weight and binding energy with biotin are very similar to avidin’s, so SA and biotin are often used to carry out signal amplification. The signal amplification system based on biotin–SA has the following advantages: (1) Different protein or nucleic acid molecules can bind to biotin or SA, respectively. Since one SA can bind to four biotins, and one protein or nucleic acid molecule can bind to multiple biotins, the effect of multi-stage amplification can be achieved. (2) The binding ability of biotin and SA is very strong, at 10^5^–10^6^ times antigen antibody affinity. This strong combination can be formed in a very short time. (3) Various types of molecules, such as proteins, enzymes, DNA, and antibodies, can bind to biotin or SA. Their biological activities do not change after binding. Therefore, different biomolecules can be modified with biotin or SA according to the specific needs of detection to achieve the corresponding signal amplification effect [79].

**Figure 9:**
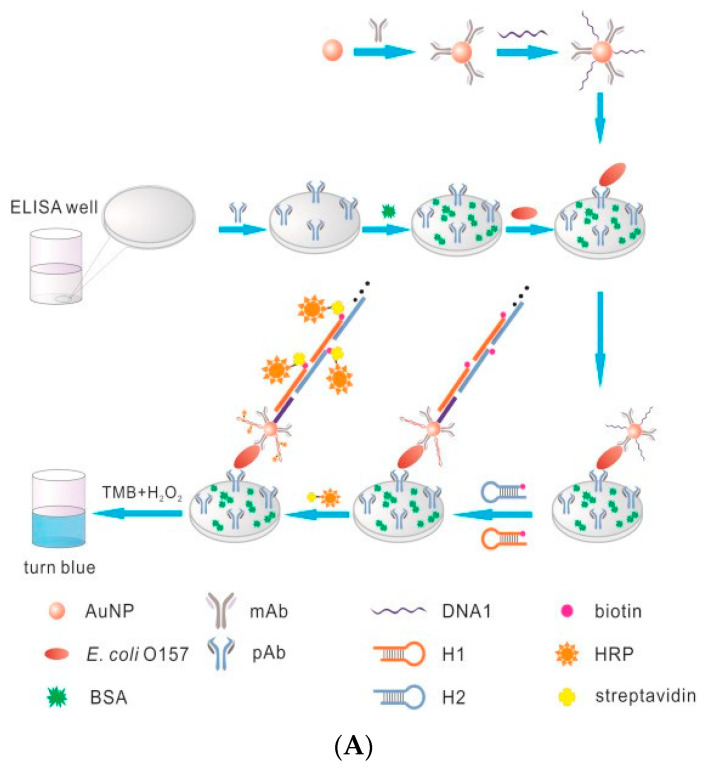

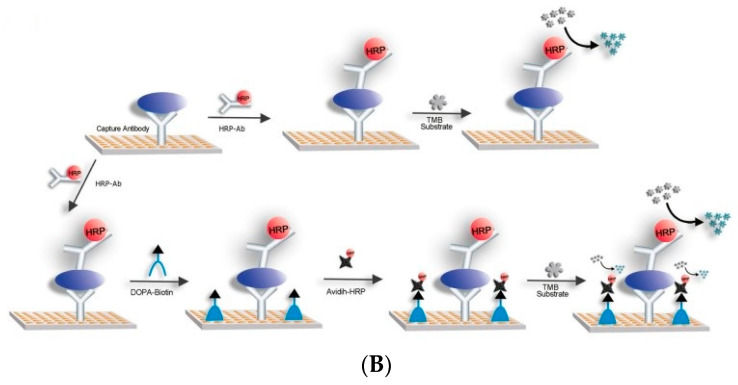
Application of biotin–SA-based signal amplification technology in the detection of foodborne pathogens: (**A**) *E. coli* O157:H7 detection [85]; (**B**) multiple foodborne pathogens detection [86]. DOPA: dopamine.

A signal amplification system based on biotin–avidin has many advantages, and so, it is widely used in clinical medicine, molecular biology, and other fields [80,81,82]. For example, Hong et al. (2012) developed a novel sandwich electrochemical enzyme immunosensor that combines the biotin–avidin signal amplification system and high catalytic activity Pt nanoparticles (PtNPs) [83]. Firstly, an antibody that can recognize the target molecule is fixed on the electrode with biotin. Another antibody that could bind to the target molecule is labeled with biotin. Through the recognition of the two antibodies, a double antibody sandwich structure is formed with alpha fetoprotein (AFP). The antibody exposed on the surface of the electrode connects platinum nanoparticles to the double antibody sandwich structure through biotin–avidin binding. A large amount of HRP, which can significantly enhance the current response, is loaded on platinum nanoparticles to realize the amplification of electrical signals. The signal amplification system reduces the detection limit of AFP to 0.08 ng/mL. Cheng et al. (2010) combined RCA technology, oligonucleotide functionalized QDs, and the biotin–SA signal amplification system to detect ultra-low concentration protein targets by anodic stripping voltammetry [84]. Through the biotin–SA signal amplification system, one SA molecule can combine with a large number of signal molecules, thus greatly enhancing the electrical signal. This method can realize the ultra-sensitive detection of human vascular endothelial growth factor, and the detection range is as low as 1 aM to 1 pM. This effect shows that the biotin–SA signal amplification system can be a powerful tool for proteomics research and clinical diagnosis. In the detection and analysis of foodborne pathogens, the biotin–SA signal amplification system has also been widely used. For example, Guo et al. (2016) designed a new ELISA technology by combining HCR technology with the biotin–SA signal amplification system [85]. In this technology, the target bacteria *E. coli* O157:H7 was used to form a double antibody sandwich structure using two recognition antibodies. One side of the antibody was modified on the surface of colloidal gold particles. The colloidal gold particles were also labeled with HCR hairpin probes, and each hairpin probe was connected with HRP. When the HCR reaction was triggered, a large amount of HRP was loaded on the double antibody sandwich, which then catalyzed the color development of TMB to cause more obvious color changes. After that, the absorbance test could be implemented. The optical biosensor based on the signal amplification system can detect amounts of *E. coli* O157:H7 as low as 1.08 × 10^2^ CFU/mL. Compared with the traditional ELISA method, the detection limit is nearly 185 times lower, and the sensitivity is improved (Figure 9A). Wan et al. (2017) developed a new ELISA method by combining dopamine (DOPA) and the biotin–SA signal amplification system, which can load more HRP than traditional ELISA technology [86]. The color change caused by TMB was more obvious, and 1.5 × 10^2^ CFU/mL of bacteria could be detected by naked eye observation (Figure 9B).

### 3.4. Signal Amplification Technology Based on Click Chemistry

Click chemistry was first proposed by Kolb (2001) in 2001 [87]. It is a kind of chemical synthesis reaction that can quickly and reliably form different kinds of molecules through the splicing of each small unit. Click chemistry, as with most other biorthogonal reactions, has high specificity. At room temperature, the reaction can take place quickly in water without any by-products. Therefore, compared with the conventional reaction mode, high selectivity and high efficiency are the most prominent advantages of click chemistry. It provides control means and flexibility for manipulating biological systems with high selectivity and rapid biological reactions [88]. In addition, because the ligands of click chemistry are all small molecules, they can be easily coupled with biological macromolecules such as proteins, antibodies, enzymes, and nanoparticles to achieve signal amplification. The size of ligands in click chemistry is very small, and a chemical complexation process using click chemistry will not interfere with the biological activity of labeled molecules. Based on these advantages of click chemistry, it is widely used in biosensors with different functions. At present, there are four types of click chemistry: cycloaddition (1,3-dipolar cycloaddition and copper free cycloaddition catalyzed by copper (I) of azides and alkynes) [89], the nucleophilic ring opening reaction, the carbonyl reaction of non-aldehydes, and the addition reaction of carbon–carbon multiple bonds. Among them, the Cu (I)-catalyzed cycloaddition of alkynyl and azide is the most widely used reaction.

**Figure 10:**
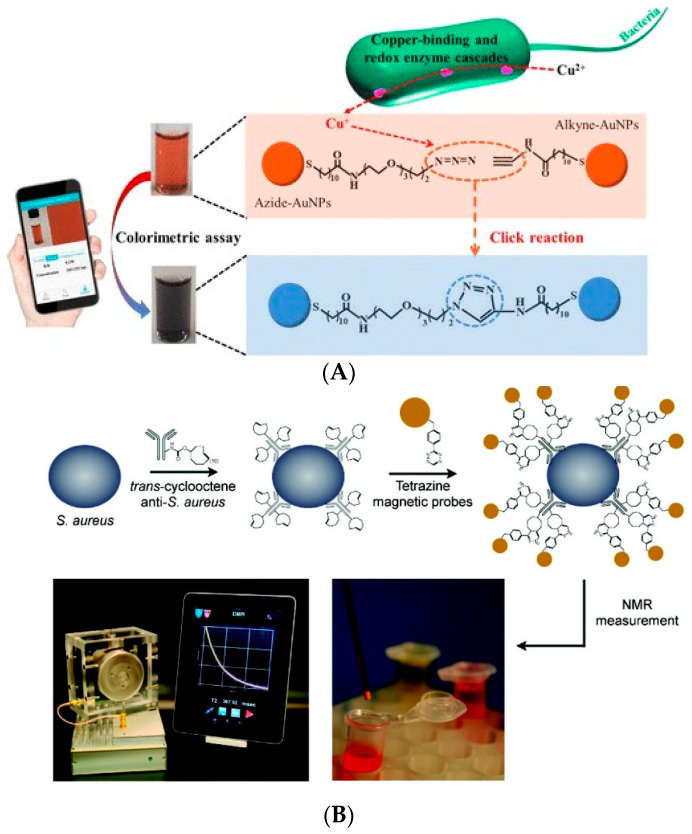
Application of click chemistry based on signal amplification technology for the detection of foodborne pathogens: (**A**) *E. coli* detection [96]; (**B**) *S. aureus* detection [97].

Combined with the signal amplification system of click chemistry, the detection sensitivity of biosensors can be effectively improved. These sensors are often used in the fields of medical diagnosis, food safety, and biological processing [90,91,92,93,94]. For example, Xianyu et al. (2018) introduced 1,2,4,5-tetrazine (TZ) and trans-cyclooctene (TCO) to polymerize single HRPs into an HRP polymer [95]. The polymers were coupled with different kinds of targets to form a double antibody sandwich, and multiple detection was carried out on the designed microfluidic chip. A large amount of HRP enzyme loaded on the double antibody sandwich can greatly improve the detection signal. The biosensor can simultaneously detect interleukin-6, procalcitonin, and C-reactive protein with detection limits as low as 0.47 pg/mL, 2.6 pg/mL, and 40 ng/mL, respectively. Click chemistry is widely used in the detection of foodborne pathogens. For example, Mou et al. (2019) used pathogenic bacteria to capture and reduce exogenous Cu^2+^ to Cu^+^ through the metabolic process [96]. The resulting Cu^+^ can trigger the click chemical reaction between the azides modified on the surface of colloidal gold particles and alkyne functional molecules. This leads to the aggregation of monodisperse colloidal gold particles, resulting in color changes. This method can detect 40 CFU/mL *E. coli* within 1 h, which verifies the signal amplification effect of click chemistry (Figure 10A). Liong et al. (2011) modified TZ and TCO on the antibody of *S. aureus* [97]. This leads to the connection of a large number of magnetic nanoparticles on the bacterial surface. By amplifying the magnetic signal, the click chemistry system can detect the concentration of *S. aureus* as low as 2 × 10^2^ CFU/mL (Figure 10B).

### 3.5. Signal Amplification Technology Based on Cascade Reaction

Cascade reaction refers to the simultaneous triggering of multiple reactions by some intermediate medium in the same reaction system. This reaction mode can achieve the following effects: (1) triggering a cascade reaction on the basis of a signal generated by a single reaction can further enhance and amplify the signal; (2) converting a single reaction that cannot trigger signal conversion into a detectable signal through a cascade reaction; and (3) through the cascade reaction, the signal generated in the original single reaction mode is converted into another signal with higher sensitivity for output to achieve the purpose of signal amplification [98,99]. At present, the multi-enzyme catalytic reaction system and signal output conversion system are often used in the cascade reaction for signal amplification. Combined with biosensors, they are applied to detection and analysis in various fields, such as food safety, bioassay, environmental monitoring, and clinical medicine [100,101,102,103].

**Figure 11:**
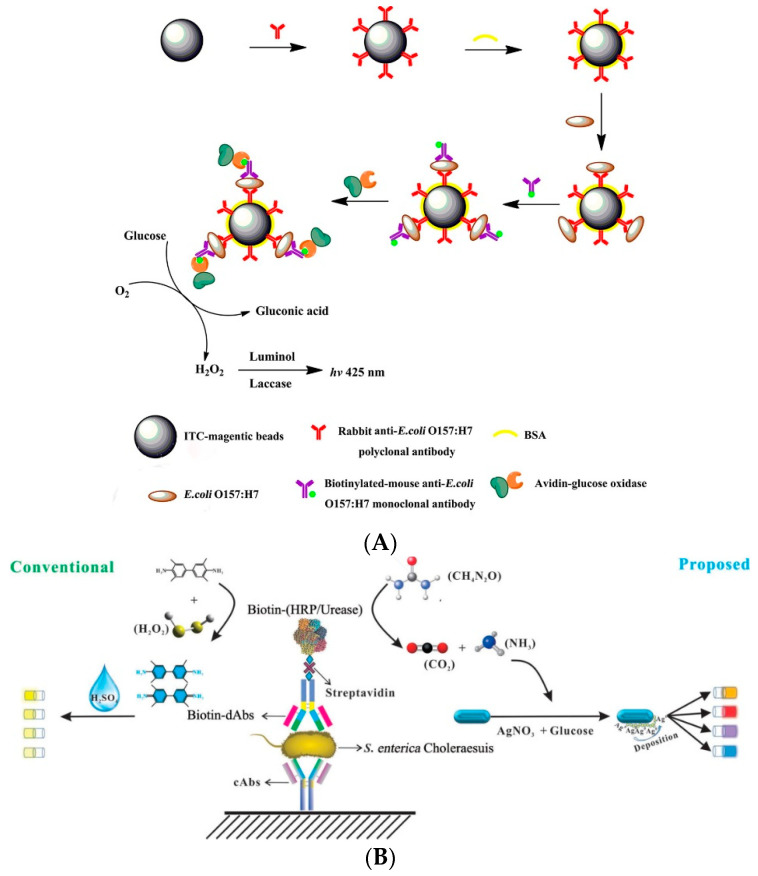
Application of cascade reaction-based signal amplification technology for the detection of foodborne pathogens: (**A**) *E. coli* O157:H7 detection [105]; (**B**) *S. typhimurium* detection [106].

Xiang et al. (2016) prepared DNAzyme hydrogel using the DNA structural unit of enzymatic polymerization. A mixed cascade enzymatic reaction system was constructed by encapsulated GOx and ß-Galactosidase (ß-Gal) in the DNAzyme hydrogel [104]. ß-Gal can convert lactose into glucose, and the glucose produced by this catalytic reaction can be catalyzed by GOx to produce gluconic acid and H_2_O_2_. DNAzyme hydrogels with mimic enzyme properties can also transform ABTS^-^ into ABTS^2-^ in the presence of H_2_O_2_, resulting in color changes. This method allows the detection of lactose at levels less than 2 mM with the naked eye, which shows that multi-enzyme catalysis can achieve a good signal amplification effect. Zhang et al. (2014) applied the multi-enzyme catalytic system to a chemiluminescence biosensor for the signal amplification detection of *E. coli* O157:H7 [105]. This uses a capture antibody and recognition antibody to form a double antibody sandwich with magnetic nanoparticles, *E. coli* and GOx. The GOx on the complex can catalyze glucose to produce H_2_O_2_. H_2_O_2_ can be hydrolyzed by lactase to induce luminol luminescence to achieve the detection of *E. coli* O157:H7. The multi-enzyme catalytic system can avoid the influence of the accumulation of catalytic products on the enzyme activity. Thus, the sensitivity of the detection method can be further improved. Under optimal reaction conditions, the detection limit of the multi-enzyme catalytic system for *E. coli* O157:H7 was 1.2 × 10^3^ CFU/mL (Figure 11A). Gao et al. (2019) developed an ELISA method based on cascade reaction signal amplification technology to compare urease and HRP [106]. The ELISA method can capture and recognize *S. typhimurium* with the double antibody sandwich mode. HRP and urease were modified on the recognition antibody to compare the results. HRP catalyzes the color reaction of TMB, through which *S. typhimurium* can be detected. In the presence of silver nitrate and glucose, the ammonia produced by urease can generate silver on the surface of gold nanorods. Different amounts of silver lead to different colors of gold nanoparticles. *S. typhimurium* can be detected by the absorbance detection of the color change. The results showed that the cascade reaction method based on urease catalysis allowed the detection of 1.21 × 10^2^ CFU/mL of *S. typhimurium* with the naked eye, while concentration as low as 1.21 × 10^1^ CFU/mL could be detected by absorbance measurement. The sensitivity of this method is two to three orders of magnitude higher than that of the HRP-based catalytic method, indicating that the cascade reaction-based biosensor has a signal amplification function (Figure 11B).

### 3.6. Signal Amplification Technologies Based on Nanomaterials

Nanomaterials refer to materials with at least one dimension in the nanometer scale (0.1–100 nm) in three-dimensional space or composed of these materials as basic units. This leads to unique functions and properties in terms of chemistry, biology, physics, mechanics, optics, magnetism, and electricity [6,107]. The application of nanomaterials in biosensors for signal amplification is mainly based on the following characteristics: (1) nanomaterials have a large specific surface area and surface free energy, so they can load a large number of signal molecules to achieve signal amplification; (2) many nanomaterials have good conductivity, and when used in electrochemical biosensors, they help to accelerate the conduction of electrons and shorten the signal response time; (3) nanomaterials have good biocompatibility, which can immobilize biomolecules without affecting their activity and maintain the stability of biosensors; (4) many nanomaterials show mimic enzyme activity, meaning that they can specifically catalyze the signal conversion of certain substrates, have fewer requirements for the external environment, and are more stable than biological enzymes. With these advantages, nanomaterials are widely used in food safety, medical diagnosis, drug analysis, and environmental monitoring fields. According to the structure of nanomaterials, they can be divided into zero dimensional, one-dimensional, two-dimensional, and three-dimensional materials. The zero-dimensional nanomaterials are mainly nanoparticles, such as colloidal gold particles, QDs, and so on. One-dimensional nanomaterials mainly include nanorods, nanowires, and nanotubes [108]. Two-dimensional nanomaterials mainly have a thin film structure; the typical representative is graphene film [109]. As the name suggests, three-dimensional nanomaterials have a three-dimensional structure, which are mainly flower like, circular porous, and dendritic [110]. At present, there are mainly four types of nanomaterials used for signal amplification: (1) nanomaterials with good optical absorption, fluorescence, electrochemical properties, and a large specific surface area, which can be directly used as signal markers of biosensors, such as colloidal gold particles, QDs, and so on; (2) nanomaterials that can load and release a large number of signal molecules, such as MnO_2_ nanosheets and mesoporous materials; (3) nanomaterials that can load and enhance the strength of many signal molecules, such as nanoflowers; (4) nanomaterials that have simulated enzyme catalytic activity and can load a large number of signal molecules, such as metal–organic frameworks. In this paper, the signal amplification function of nanoflowers, mesoporous materials, and metal–organic frameworks (MOFs) in biosensors is reviewed. The typical shapes of nanoflowers, mesoporous materials, and MOFs are presented in Table 1.

The full name of nanoflowers is organic/inorganic hybrid nanoflowers (NFs). From the definition, it can be seen that this refers to a nanomaterial with a flower-shape, which is formed by the self-assembly of inorganic salt ions and organic ligands (such as proteins, enzymes, and antibodies). The first successful preparation of nanoflowers was carried out in 2012 by Ge et al. (2012) [111]. They used copper phosphate as an inorganic salt ion and different kinds of proteins (bovine serum albumin, BSA, etc.) as organic ligands. After that, nanoflowers have been increasingly studied and reported, and their unique properties are constantly being discovered. At present, the size of nanoflowers is relatively large (0.5–2 μm), with a large specific surface area that can load a large number of signal molecules. At the same time, it has been found that nanoflowers can improve the activity of biomolecules. One of the most common functions is to improve the catalytic activity of biological enzymes. Therefore, as a medium of signal amplification, nanoflowers have great application prospects in the field of biosensors.

**Figure 12:**
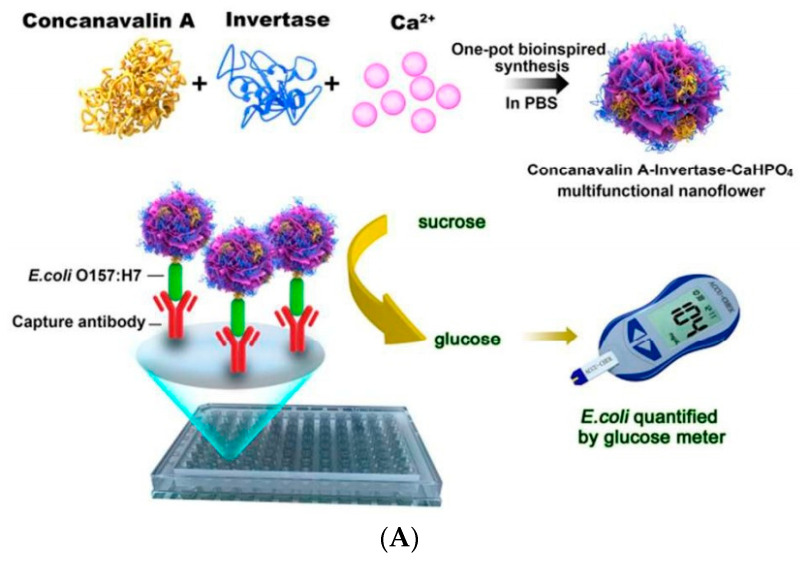

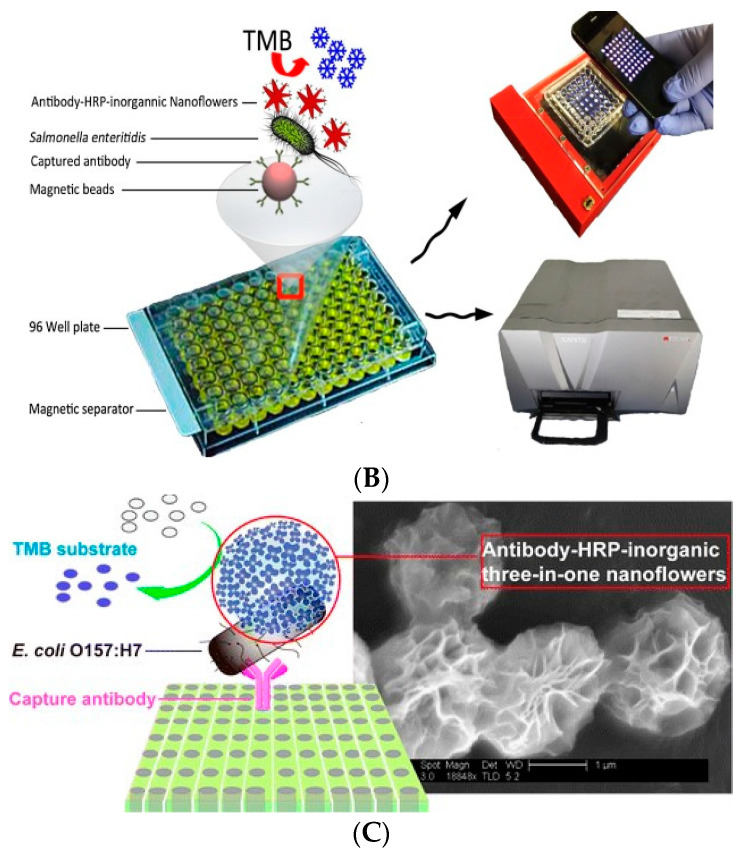
Application of nanoflower-based signal amplification technology for the detection of foodborne pathogens: (**A**) [112] and (**C**) [114] *E. coli* O157:H7 detection; (**B**) *S. enteritidis* detection [113].

Due to the relatively large size of nanoflowers, they can easily form a complex with bacteria, which is very beneficial for analysis and detection. Their application in the rapid and sensitive detection of foodborne pathogens has been widely reported. For example, Ye et al. (2016) prepared nanoflowers with ConA (which can recognize the target bacteria), sucrose invertase, and inorganic calcium ions [112]. An antibody that can capture *E. coli* O157:H7 is immobilized on the ELISA plate, and the nanoflowers are added to the ELISA plate to detect the target bacteria. The concentration of *E. coli* O157:H7 as low as 10^1^ CFU/mL could be detected by measuring the concentration of glucose produced by invertase transforming sucrose with a blood glucose meter (Figure 12A). Maarouf et al. (2018) prepared nanoflower materials that can bind to *S. enteritidis* by mixing antibodies with HRP and inorganic calcium ions, respectively, with which the antibodies can recognize *S. enteritidis* [113]. The double antibody sandwich was formed by nanoflowers, magnetic beads, and *S. enteritidis*. The HRP on this structure catalyzes the color reaction of TMB. Using a smartphone application to compare the solution color, this method is able to detect the target bacteria conveniently (Figure 12B). This method can detect concentrations of *S. enteritidis* as low as 1.0 CFU/mL, which indicates that the signal amplification technology based on nanoflowers can quickly and easily screen foodborne pathogens. Wei et al. (2016) constructed an ELISA optical biosensor based on nanoflower signal amplification for the rapid and sensitive detection of *E. coli* O157:H7 [114]. The nanoflowers were prepared from recognition antibody, HRP, and copper phosphate. The HRP catalyzed TMB to generate a color change that can be detected by absorbance. This method could detect concentrations of *E. coli* O157:H7 as low as 60 CFU/mL (Figure 12C).

**Figure 13:**
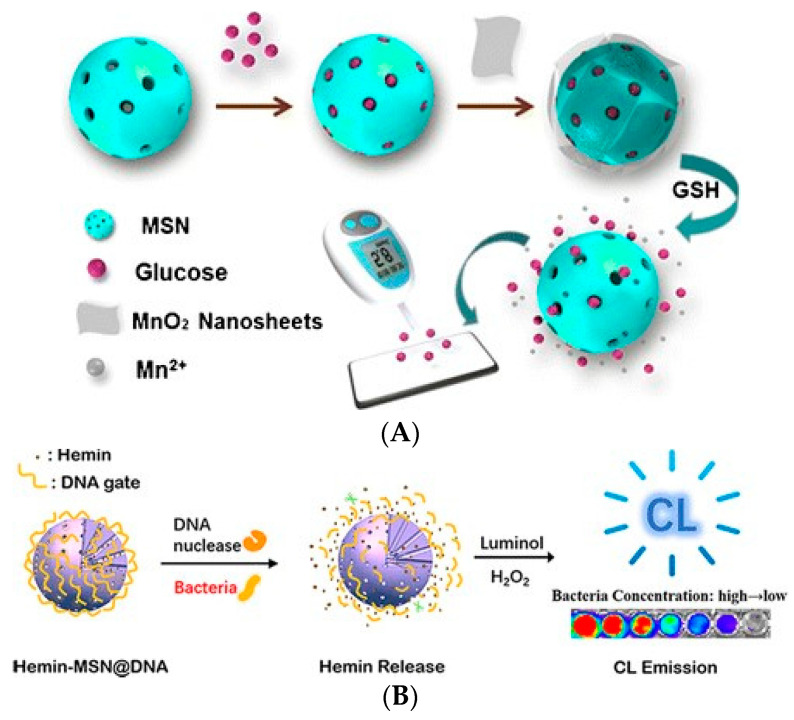
Application of mesoporous nanoparticle-based signal amplification technology for rapid detection: (**A**) GSH detection [118]; (**B**) *E. coli* O157:H7 and *S. aureus* detection [119].

Mesoporous nanoparticles are a kind of porous material with pore sizes between 2 and 50 nm. Since the pore size covers a range of particle sizes from small biological molecules to proteins and other macromolecules, it can be used as an excellent carrier for various guest molecules [115,116]. Mesoporous materials have the following advantages, which make them play a very important role in signal amplification technology: (1) mesoporous materials have a large specific surface area, which can load a number of signal molecules; (2) the pore size and shape of mesoporous materials can be adjusted by changing the preparation conditions to meet the loading requirements of different kinds of signal molecules; (3) by introducing other substances, the mesoporous property can be blocked to avoid the early release of signal molecules, so as to maximize the signal intensity [117]. At present, mesoporous carbon nanoparticles (MCNs) and mesoporous silica nanoparticles (MSNs) are the most common mesoporous materials. The first step of signal amplification based on mesoporous materials is to load the signal molecules. Then, some molecules or materials are used to seal the mesoporous material to prevent the loss of signal molecules. Finally, a substance (or the target itself) is introduced to disintegrate the sealing material and release the signal molecules. By monitoring the signal molecules, the quantitative analysis of the analytes can be achieved. For example, Tan et al. (2018) loaded a large number of glucose molecules into MSNs [118]. Then the prepared MnO_2_ nanosheets were used to block all the mesopores of MSNs. Finally, glutathione (GSH) was introduced to decompose the nanosheets to release glucose. The linear relationship between glucose concentration and GSH can be obtained by detecting glucose molecules with a blood glucose meter. The lowest detectable concentration of GSH was 34 nM/mL (Figure 13A). Gu et al. (2019) loaded a large amount of heme in MSNs. The mesopores of MSNs were encapsulated with DNA strands, which could be specifically recognized by nuclease or bacterial lysate [119]. In the presence of the target, the DNA strand wrapped on the surface of MSNs was stripped off from MSNs, releasing a number of heme molecules that could cause luminol luminescence. When the system was triggered by *E. coli* O157:H7 and *S. aureus*, the lowest detection limits were 3.0 CFU/mL and 2.5 CFU/mL, respectively (Figure 13B).

**Figure 14:**
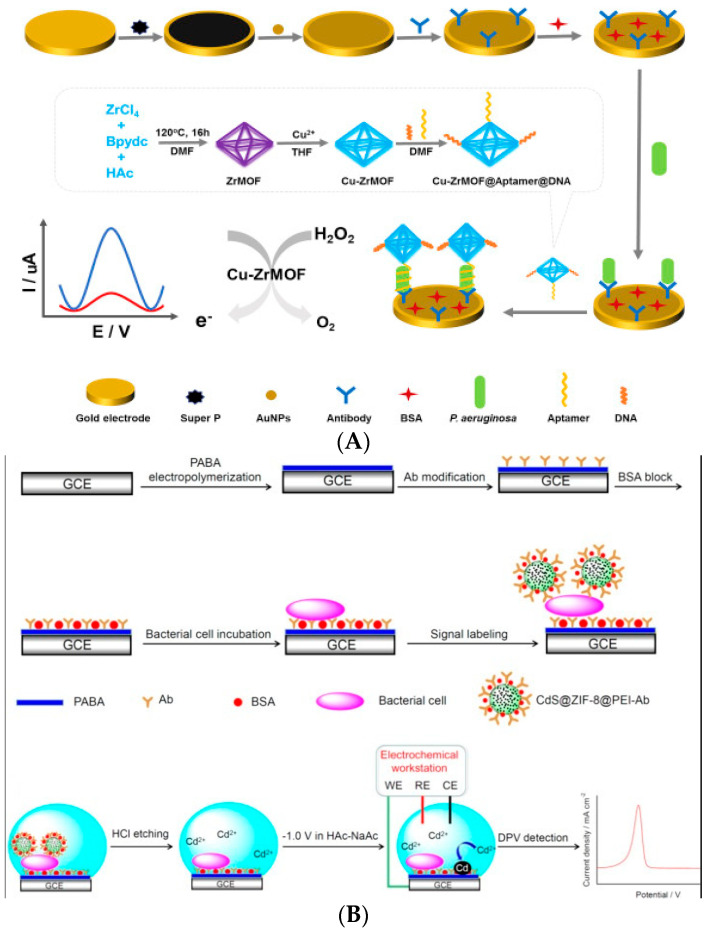
Application of metal–organic frameworks (MOFs)-based signal amplification technology for the detection of foodborne pathogens: (**A**) *Pseudomonas* spp. detection [125]; (**B**) *E. coli* O157:H7 detection [126].

Metal–organic frameworks (MOFs) usually refer to crystal materials with a grid structure formed by the self-assembly of inorganic metals and organic ligands [120]. Compared with traditional porous materials, the biggest advantage of MOFs is that different crystal structures can be prepared by changing the coordination number of metal centers and the size of organic ligands [121]. In recent years, MOFs have developed rapidly. MOFs with different structures and properties were designed, synthesized, and introduced into biosensors for signal amplification [122]. In biosensors, MOFs can achieve signal amplification in the following ways. (1) Most MOFs have good conductivity and can be used in electrochemical sensors to enhance the electrical signal. (2) Some MOFs have mimic enzyme activity and can catalyze the signal conversion of specific substrates. Compared with biological enzymes, these have the advantages of better stability and lower cost. (3) The porous lattice structure of MOFs has a large specific surface area, which can be used to load a large number of signal molecules to achieve a signal amplification effect [123]. As a result of these advantages, MOFs have been used in the detection of foodborne pathogens. For example, Shahrokhian et al. (2018) developed an electrochemical biosensor based on amino MOFs for the detection of *E. coli* O157:H7. Based on their good conductivity, MOFs were modified with amino groups and fixed on the electrode surface to enhance the electrochemical activity of the electrode [124]. Subsequently, *E. coli* O157:H7 was captured on the surface of MOFs by aptamers. Finally, methylene blue was used as an electrochemical indicator, and differential pulse voltammetry was used to detect *E. coli* O157:H7. Using the current change as the signal for analysis, the concentration could be detected at levels as low as 2 CFU/mL. Zhang et al. (2019) prepared Cu-ZrMOF with high catalytic activity, which has mimic enzyme activity and can catalyze the decomposition of H_2_O_2_ [125]. An antibody that can recognize *Pseudomonas aeruginosa* was modified on the electrode surface to capture the target bacteria. The Cu-ZrMOF labeled with aptamer can combine with *Pseudomonas* spp. to form a complex. The Cu-ZrMOF on the complex can catalyze H_2_O_2_-induced electron transfer. Using cyclic voltammetry, the target bacteria can be quantified by the electric signal intensity. This method can detect concentrations of *Pseudomonas* spp. as low as 2 CFU/mL (Figure 14A). Zhong et al. (2019) prepared the Zeolitic Imidazolate Framework-8 (ZIF-8), which can encapsulate CdS QDs (CdS QDs) and form CdS@ZIF-8 [126]. Taking advantage of the large specific surface area, it could load a large number of signal molecules. An antibody that could capture *E. coli* O157:H7 was modified on the electrode. The antibody on CdS@ZIF-8 could recognize the target bacteria and form a double antibody sandwich complex. CdS@ZIF-8 was decomposed by adding hydrochloric acid solution to release a large amount of Cd (II) ions. By measuring the released Cd (II) ions by differential pulse voltammetry, *E. coli* O157:H7 can be quantitatively detected. The minimum detection limit of this method is 3 CFU/mL (Figure 14B).

In summary, different kinds of signal amplification technologies have been widely used and studied in various fields. For food safety detection, the application of signal amplification technology in biosensors can improve stability, simplify the detection steps, and reduce costs. Further innovation and work on the basis of existing signal amplification technologies will occur in the future with the aim of building biosensor platforms with a high stability, convenience, and low cost and that are better able to solve the problems of food safety.

### 3.7. Signal Processing Technologies Using Deep Learning

Processing signals generated in the detection is an important part of biosensors. The signals can be in different forms, such as images or spectra, and the identification of foodborne pathogens is sometimes time consuming. In recent years, deep learning technology has begun to appear in biosensors for signal enrichment and rapid processing. For example, in the processing of spectra, deep learning has the potential to enhance the output of in-line, on-line, and at-line instrumentation used for process analytical technology in biosensing.

Wang et al. (2020) presented a computational live bacteria detection system that periodically captures coherent microscopy images of bacterial growth inside a 60-mm-diameter agar plate [127]. It analyzed these time-lapsed holograms using deep neural networks for the rapid detection of bacterial growth and the classification of the corresponding species.

**Figure 15:**
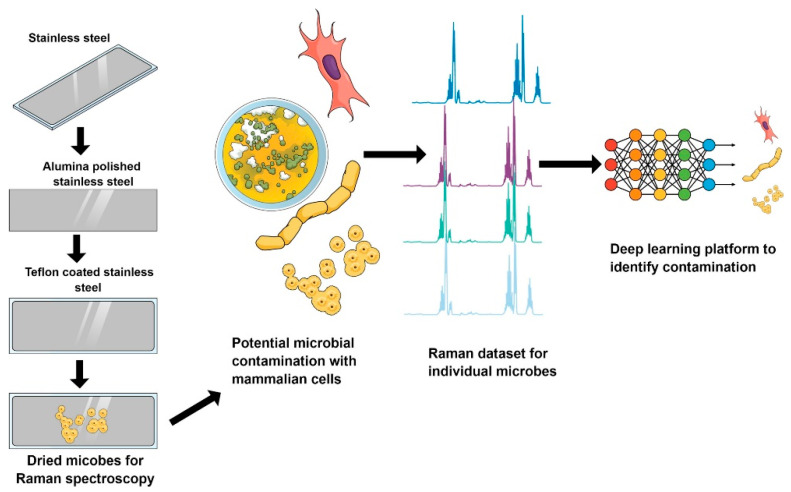
Detecting microbial contamination using Raman spectroscopy-based deep learning strategies [128].

Maruthamuthu et al. (2020) developed a tool for detecting microbial contamination using Raman spectroscopy-based deep learning strategies [128]. A Raman dataset of microorganisms were built to train a convolution neural network (CNN). The dataset contains common contaminants in Chinese Hamster Ovary (CHO) cells. This kind of cells is often used in the pharmaceutical industry. The dataset of 12 microbes spans across Gram-positive and Gram-negative bacteria as well as fungi. The trained network classified the different samples comprising individual microbes and microbes mixed with CHO cells with an accuracy of 95–100%. An attention map was also created for different microbes and CHO cells to highlight which segments of the Raman spectra contribute the most to help discriminate between different species. The dataset and model provide a route for implementing Raman spectroscopy for detecting pathogens in the production of biologics (Figure 15).

Kukula et al. (2021) presented a deep learning-based approach to detect the identity of a bacteria class rapidly and accurately [129]. The model was driven by a Raman spectroscopy dataset. They used a four-layer CNN architecture and a 30-class bacteria isolate dataset for training and testing. The identification accuracy was around 86% with speeds close to real time. This optical/biological detection method is promising for applications in the detection of microbes in liquid biopsies and concentrated environmental liquid samples, where fast and accurate detection is crucial.

Yan et al. (2021) proposed a machine learning strategy based on fingerprint difference of Raman spectroscopy for the rapid diagnosis of pathogenic bacteria [130]. In their research, 15,890 single-cell Raman spectra of 23 common strains from seven genera were collected at the single cell level. The nonlinear features of raw data were extracted by kernel principal component analysis, and the individual bacterial cell was evaluated and discriminated at the serotype level through the decision tree algorithm. Four-level classification models were introduced, and the different hierarchies of the identification models achieved accuracies in the range of 87.1–95.8%. This method realized the efficient prediction of strains at the serotype level.

Maruthamuthu et al. (2020) summarized a series of process analytical technologies (PAT) that applied data-driven deep learning [131]. PAT for the manufacture of monoclonal antibodies is defined by an integrated set of advanced and automated methods. These methods process monitoring and soft sensors to detect microbial or mycoplasma contamination. They analyze the compositions and biophysical properties of cell culture fluids, cell-free product streams, and biotherapeutic molecules that are ultimately formulated into concentrated products. The implementation of PAT for the development and manufacture of mAbs is now gaining momentum with pilot-scale demonstrations of multiattribute monitoring and potential for process control. Maruthamuthu et al. (2020) sufficiently reviewed the current status of mAb manufacturing, associated challenges, and how PAT and data analytics can help overcome these challenges to develop a new therapeutic product [131].

### 3.8. Summary

In order to better understand the advantages and disadvantages of the reported biosensors, a general comparison on incubation time, detection limit, and detection range for different types of biosensors and different signal amplification methods is summarized and shown in Table 2. Among different types of biosensors, the impedimetric biosensor reported by Farka et al. (2016) had the shortest detection time of 20 min, and the fluorescent biosensor reported by Krishnan et al. (2014) showed the lowest detection limit of 5 CFU/mL for *E. coli* [24,46]. Among these signal amplification methods, the enzymatic catalysis based on the one reported by Qiao et al. (2017) had the shortest detection time of 6 min, and the nanomaterials (nanoflowers, mesoporous materials, and metal–organic frameworks) based ones reported by Gu et al. (2019), Shahrokhian et al. (2018), Zhang et al. (2019), and Zhong et al. (2019) showed 100 CFU/mL for different bacteria [66,119,124,125,126].

## 4. Conclusions and Future Trends

Foodborne pathogens pose a great threat to the economy, environment, and human health. The early screening of foodborne pathogens is of great significance for food safety. This paper reviews current detection technologies, mainly regarding biosensors coupled with signal amplification technology. The advantages and disadvantages of different kinds of biosensors and signal amplification technologies were analyzed, providing a comprehensive reference for the sensitive and rapid detection of foodborne pathogens. In addition, the biosensors and signal amplification technologies introduced in this article have been affected by other factors (such as human operation, detection equipment, and environmental interference), resulting in the detection time and sensitivity being different in different studies. Thus, the readers can choose the best suitable biosensors or signal amplification technologies according to their own needs without being limited by the detection sensitivity and time mentioned in this article.

To date, many researchers have devoted efforts to the development and innovation of sensitive detection methods for pathogenic bacteria. However, the food background is usually complex and easily disrupted by other non-target substances, non-specific proteins, and so on. In addition, the concentration of bacteria in the screening samples is generally low, which brings great challenges to the screening of pathogenic bacteria. Thus, the pre-processing of large-volume and complex food samples is of great significance for the rapid and sensitive detection of bacteria. While applying biosensors and signal amplification technologies, the introduction of efficient bacteria separation and enrichment technology in large-volume samples will be an important trend of bacterial detection in the future.

As a multidisciplinary high-tech field, the future development of the biosensor will have some new characteristics with the rapid development of biological sciences, information sciences, and materials sciences. First, biosensors may develop toward miniaturization and comprehensiveness. Future biosensors will further involve various fields of healthcare, food inspection, environmental monitoring, and the fermentation industry. With the advancement of micro-processing technology and nanotechnology, biosensors will continue to be miniaturized. Second, in the future, biosensors will be perfectly and closely integrated with computers, which can automatically collect and process data, providing more scientific and accurate results. At the same time, microfluidic technology will increasingly enter the field of biosensors to realize the integration and integration of detection systems.

We believe that with the further improvement of some key technologies including signal enrichment and analysis, and with the continuous development of various disciplines, biosensors will surely be more powerful in the future.

## Figures and Tables

**Table 1:** The shapes of nanoflowers, mesoporous materials, and MOFs.

Nanoflowers	Mesoporous Materials	MOFs
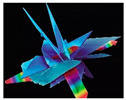	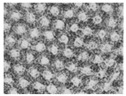	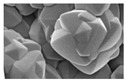
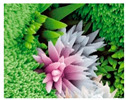	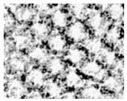	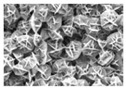
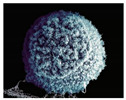	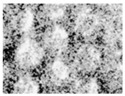	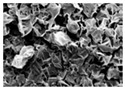

**Table 2:** A comparison of different types of biosensors and different signal amplification methods.

		Bacterial Species	Incubation Time	Detection Limit	Linear Range	Reference
Biosensors	Impedimetric biosensors	*S. typhimurium*	-	1 × 10^3^ CFU/mL	10^3^–10^8^ CFU/mL	[24]
		*E. coli*	30 min	3 × 10^1^ CFU/mL	10^1^–10^8^ CFU/mL	[25]
		*L. monocytogenes*	75 min	1.6 × 10^3^ CFU/mL	1.9 × 10^3^–1.9 × 10^6^ CFU/mL	[26]
	Amperometric biosensors	*E. coli* O157:H7	10 min	10^2^ CFU/mL	-	[30]
		*S. enteritidis*		10^2^ CFU/mL	-	
		*L. monocytogenes*		10^2^ CFU/mL	-	
		*E. coli* O157:H7	15 min	30 CFU/mL	3 × 10^1^–3 × 10^7^ CFU/mL	[31]
		*E. coli* O157:H7	45 min	10^2^ CFU/mL	10^2^–10^5^ CFU/mL	[32]
	Colorimetric biosensors	*S. typhimurium*	45 min	10 CFU/mL	25–10^5^ CFU/mL	[38]
		*S. typhimurium*	45min	10^2^ CFU/mL	10^2^–10^5^ CFU/mL	[39]
		*L. monocytogenes*	45 min	10^2^ CFU/mL	1.1 × 10^2^–1.1 × 10^6^ CFU/mL	[40]
	Fluorescent biosensors	*E. coli*	60 min	5 CFU/mL	10–10^2^ CFU/mL	[46]
		*S* *. typhimurium*	30 min	5 × 10^2^ CFU/mL	2.5 × 10^3^–1.95 × 10^8^ CFU/mL	[47]
		*E. coli* O157:H7	2.5 h	8 × 10^1^ CFU/mL	10^1^–10^4^ CFU/mL	[48]
		*S. aureus*		10^2^ CFU/mL	10^1^–10^4^ CFU/mL	
		*L. monocytogenes*		4.7 × 10^1^ CFU/mL	10^1^–10^4^ CFU/mL	
		*S. typhimurium*		1.6 × 10^2^ CFU/mL	10^1^–10^4^ CFU/mL	
	Microfluidic biosensor	*E. coli*	25 min	10^3^ CFU/mL	10^3^–10^5^ CFU/mL	[57]
		*E. coli*	-	3 × 10^2^ CFU/mL	3 × 10^2^–3 × 10^6^ CFU/mL	[58]
		*S. typhimurium*	30 min	10^3^ CFU/mL	-	[59]
		*E. coli*	10 min	5.4 × 10^3^ CFU/mL	10^4^–10^6^ CFU/mL	[55]
Signal amplification methods for biosensors	Based on enzymatic catalysis	*E. coli* O157:H7	-	12 CFU/mL	10–10^7^ CFU/mL	[66]
		*S. aureus*	30 min	11 CFU/100 μL	5 × 10^2^–5 × 10^4^ CFU/mL	[67]
		*S. typhimurium*	45 min	72 CFU/mL	1.27 × 10^2^–1.27 × 10^5^ CFU/mL	[68]
	Based on nucleic acid amplification	*S. aureus*	6 min	4 × 10^2^ CFU/mL	50 pM–100 nM	[77]
		*V. parahaemolyticus*	50 min	10 CFU/mL	10-10^6^ CFU/mL	[71]
		*E. coli* O157:H7	60 min	34 CFU/mL	3.7 × 10^1^–3.7 × 10^7^ CFU/mL	[78]
		*S. typhimurium*		6.4 CFU/mL	3.0 × 10^1^–3.0 × 10^7^ CFU/mL	
		*L. monocytogenes*		70 CFU/mL	3.2 × 10^1^–3.2 × 10^7^ CFU/mL	
		*L. monocytogenes*	60 min	4.6 × 10^2^ CFU/mL	4.6 × 10^2^–4.6 × 10^7^ CFU/mL	[72]
	Based on biotin–streptavidin binding	alpha fetoprotein	10 min	0.08 ng/mL	0.25–100 ng/mL	[83]
		Human vascular endothelial growth factor	-	-	1 aM–1 pM/100μL	[84]
		*E. coli* O157:H7	60 min	1.08 × 10^2^ CFU/mL	5 × 10^2^–1 × 10^7^ CFU/mL	[85]
		Multiple foodborne pathogens	1 h	1.5 × 10^2^ CFU/mL	1.5×10^2^–1.5×10^7^ CFU/mL	[86]
	Based on click chemistry	Nterleukin-6	-	0.47 pg/mL	pg/mL-μg/mL	[95]
		Procalcitonin		2.6 pg/mL		
		C-reactive protein		40 ng/mL		
		*E. coli*	30 min	40 CFU/mL	10^2^–10^7^ CFU/mL	[96]
		*S. aureus*	15 min	2 × 10^2^ CFU/mL	-	[97]
	Based on cascade reaction	Lactose	30 min	2 mM	-	[104]
		*E. coli* O157:H7	-	1.2 × 10^3^ CFU/mL	-	[105]
		*S. typhimurium*	40 min–2 h	1.21 × 10^1^ CFU/mL	1.21×10^1^–1.21×10^8^ CFU/mL	[106]
	Based on nanoflowers	*E. coli* O157:H7	-	10^1^ CFU/mL	-	[112]
		*S. enteritidis*	-	1.0 CFU/mL	-	[113]
		*E. coli* O157:H7	40 min	60 CFU/mL	1.7 × 10^1^–1.7 × 10^7^ CFU/mL	[114]
	Based on mesoporous materials	Glutathione	10 min	34 nM/mL	0.1–10 μM/mL	[118]
		*E. coli* O157:H7	60 min	3.0 CFU/mL	10–10^9^ CFU/mL	[119]
		*S. aureus*		2.5 CFU/mL		
	Based on Metal-Organic Frameworks	*E. coli O157:H7*	20 min	2 CFU/mL	2.1×10^1^–2.1×10^7^ CFU/mL	[124]
		*Pseudomonas*	50 min	2 CFU/mL	10–10^6^ CFU/mL	[125]
		*E. coli* O157:H7	60 min	3 CFU/mL	10–10^8^ CFU/mL	[126]
